# Supplementary material for: Antioxidant Capacity and NF-kB-Mediated Anti-Inflammatory Activity of Six Red Uruguayan Grape Pomaces
Source: Molecules. 2023 May 5;28(9):3909. doi: 10.3390/molecules28093909 (PMC10180250; doi:10.3390/molecules28093909)
Supplement: Supplementary file 1 [file molecules-28-03909-s001.zip › molecules-2251731-supplementary.pdf]

Supplementary Materials

Table S1. Total phenolic contents (FC in mgGAE/L) and antioxidant capacity determined by DPPH, ABTS, ORAC, FRAP and ESR for the six grapes pomaces in two sample dry weights of 5 and 10 g. In all cases the extraction was made by maceration in a mixture of ethanol:water 50:50. GAE: Gallic Acid, AAS: Ascorbic Acid; TE: Trolox Equivalent. ND = not detected.

**Sample name**	**Dry Weight (g)**	**FC mgGAE/L**	**DPPH (mgAAS/L)**	**ABTS (mgAAS/L)**	**ORAC (mM TE)**	**FRAP (μg TE/mL)**	**ESR** (%)
Tannat 1	5	2747,74 ± 37,78	39.9 ± 2.2	46.00 ± 2.02	18.97 ± 2.47	133.46 ± 1.21	33.4± 1.51
Tannat 1	10	4895,60 ± 43,26	74.1 ± 4.3	67.80 ± 1.25	19.52± 3.26	305.74± 2.04	59.2± 1.67
Tannat B	5	3622,18 ± 75,47	60.0 ± 2.3	41.00 ± 1.73	22.9± 4.93	173.81± 1.37	94.9± 2.11
Tannat B	10	6045,38 ± 37,09	87.0 ± 2.7	64.10 ± 2.19	24.6± 0.14	323.58± 2.01	85.8± 1.08
Tannat 2	5	3206,63 ± 65,03	44.8 ± 7.9	35.50 ± 1.47	19.32± 5.14	152.86± 2.11	50.2± 1.13
Tannat 2	10	5234,67 ± 30,89	69.7 ± 1.1	48.30 ± 0.48	20.45± 4.03	273.92± 1.35	67.5± 2.01
Tannat 3	5	1982,92 ± 10,04	24.2 ± 1.2	42.00 ± 0.05	19.37± 0.74	45.77± 1.14	76.4± 1.17
Tannat 3	10	2696,75 ± 16,59	33.5 ± 3.8	32.80 ± 1.04	20.24± 4.81	108.63± 2,03	68.1± 1.31
Tannat 4	5	1984,19 ± 36,60	34.1 ± 8.1	32.40 ± 0.71	11.31 ± 1.21	76.56± 1.10	90.2± 1.84
Tannat 4	10	1996,94 ± 25,69	26.5 ± 3.4	37.80 ± 7.83	9.72 ± 1.01	85.35± 1.27	67.3± 1.95
Cabernet-Franc	5	436,46 ± 19,53	8.6 ± 3.9	18.40 ± 8.78	17.34± 2.74	ND	25.7± 1.07
Cabernet-Franc	10	630,21 ± 35,99	10.6 ± 7.1	17.00 ± 1.98	11.09± 1.45	3.36 ± 0.43	66.5± 1.32
